# Skeletal ligament healing using the recombinant human amelogenin protein

**DOI:** 10.1111/jcmm.12762

**Published:** 2016-02-24

**Authors:** Salem Hanhan, Ayala Ejzenberg, Koby Goren, Faris Saba, Yarden Suki, Shay Sharon, Dekel Shilo, Jacob Waxman, Elad Spitzer, Ron Shahar, Ayelet Atkins, Meir Liebergall, Anat Blumenfeld, Dan Deutsch, Amir Haze

**Affiliations:** ^1^ Faculty of Dental MedicineInstitute of Dental SciencesHebrew UniversityJerusalemIsrael; ^2^ Orthopaedic DepartmentHadassah – Hebrew University Medical CenterJerusalemIsrael; ^3^ Faculty of AgricultureKort School of Veterinary MedicineHebrew UniversityRehovotIsrael

**Keywords:** amelogenin, skeletal ligament, sport injuries, regeneration, proprioception, mesenchymal stem cells

## Abstract

Injuries to ligaments are common, painful and debilitating, causing joint instability and impaired protective proprioception sensation around the joint. Healing of torn ligaments usually fails to take place, and surgical replacement or reconstruction is required. Previously, we showed that *in vivo* application of the recombinant human amelogenin protein (rHAM ^+^) resulted in enhanced healing of the tooth‐supporting tissues. The aim of this study was to evaluate whether amelogenin might also enhance repair of skeletal ligaments. The rat knee medial collateral ligament (MCL) was chosen to prove the concept. Full thickness tear was created and various concentrations of rHAM ^+^, dissolved in propylene glycol alginate (PGA) carrier, were applied to the transected MCL. 12 weeks after transection, the mechanical properties, structure and composition of transected ligaments treated with 0.5 μg/μl rHAM ^+^ were similar to the normal un‐transected ligaments, and were much stronger, stiffer and organized than control ligaments, treated with PGA only. Furthermore, the proprioceptive free nerve endings, in the 0.5 μg/μl rHAM ^+^ treated group, were parallel to the collagen fibres similar to their arrangement in normal ligament, while in the control ligaments the free nerve endings were entrapped in the scar tissue at different directions, not parallel to the axis of the force. Four days after transection, treatment with 0.5 μg/μl rHAM ^+^ increased the amount of cells expressing mesenchymal stem cell markers at the injured site. In conclusion application of rHAM ^+^ dose dependently induced mechanical, structural and sensory healing of torn skeletal ligament. Initially the process involved recruitment and proliferation of cells expressing mesenchymal stem cell markers.

## Introduction

Ligaments play an essential role in mediating normal movement and stability of joints. Injuries of ligaments are prevalent, painful, debilitating and pose significant financial burden 1, 2. Injury often causes significant joint instability, which may lead to damage to other tissues and development of degenerative joint disease. In most cases, healing of torn ligaments fails to take place, and replacement with autologous tissues or allogeneic grafts are required. When natural healing does occur a scar tissue is formed, which lacks the unique biomechanical properties, and in a significant subset of the patients is clinically in‐sufficient 3.

Ligaments connect bones to each other to restrict their relative motion and stabilize the joints. A second and less often mentioned function of ligamentous tissue is their sensory‐motor control of joint movements. As such, ligaments are recognized as sensory organs, capable of monitoring and supplying relevant kinaesthetic and proprioceptive data 4. Mechanoreceptors, mainly free nerve endings 5, effect dynamic aspects of joint stability chiefly concerning proprioceptive control of the compressive and directional muscular forces acting on a joint. Ligaments are elastic tissues with unique composition and hierarchical structure. Their strength is related to the number and size of collagen fibrils. Ligament fibroblasts synthesize and secrete collagen [type I predominant (91%) and some type III] and other components to the extracellular matrix. Fibrillar collagen type I provides the high tensile strength of ligaments and is responsible for their hierarchical structure.

Ligaments have modest requirement for energy, which enable them to bear loads and maintain tension for extended periods of time. However, the low metabolic rate entails slow healing after injury 6. Ligaments sometimes heal naturally, but their pre‐injury structure, organization and biomechanical properties as well as their proprioceptive sensation are not restored, because of development of scar tissue. Loss of mechanical competence is mainly because of distorted composition of extracellular matrix and misalignment of collagen fibrils in the scar tissue 2. The healing process includes an inflammatory stage lasting hours to few days, a remodelling stage in which collagen type III is synthesized, synthesis of collagen I which is initiated about 6 weeks after injury, and within several months, the fibrous tissue is slowly transformed into scar tissue 6. To date, no clinical method exist which enables regeneration of injured ligaments.

Our previous studies focused on effective repair of the tooth‐supporting (periodontal) tissues, including the periodontal ligament (PDL), by application of the recombinant human amelogenin protein (rHAM^+^), produced in our laboratory 7. Periodontal ligament anchors tooth root to jaw bone, and is similar in its cellular and extracellular matrix composition to skeletal ligaments, although on the tooth side it is anchored to cementum, a thin mineralized layer covering the tooth root. We showed that *in vivo* application of rHAM^+^ alone resulted in significant and progressive repair of the tooth‐supporting tissues; alveolar bone, PDL and cementum, after induction of chronic periodontitis in the dog model 8.

Our goal was to study the effect of amelogenin on regeneration of skeletal ligaments. Injuries of the knee anterior cruciate ligament and medial collateral ligament (MCL) are very common.

In most cases, torn MCL in humans does not require clinical (surgical) intervention, although it heals by scar tissue formation and on average only 70% restoration of its pre‐injury mechanical properties is achieved. The formed tissue is sufficient to enable function due to the effect of other knee stabilizers and the normal stress the MCL encounters. However, the same biological processes occur during healing of the MCL and other extra‐articular ligaments that do not recover naturally. We chose transection of the rat MCL as our proof the concept model for regeneration of skeletal ligaments, because MCL is a superficial ligament which enables easy access, is extra‐articular and not influenced by possible effects of the synovial fluid, and is a well‐established model.

## Materials and methods

### Preparation of rHAM^+^ solutions

rHAM^+^ was produced in our laboratory, according to Taylor *et al*. 7, and stored at −80°C in 0.05 M acetic acid. The day prior to use aliquots of rHAM^+^ were lyophilized overnight. Before the operation rHAM^+^ was dissolved in propylene glycol alginate carrier (PGA) (ISP, Koln, Germany) at concentrations of 0.1, 0.5, 1 or 2.5 μg rHAM^+^/μl PGA (2–3 concentrations on each experimental day). Several concentrations of PGA in the range of 0.1–5% were tried. We found that the most appropriate concentration of PGA was 2.25%. At this concentration the PGA is viscous enough to not spill and scatter in the surrounding tissues, and its fluidity enables application of an accurate dose. All solutions were coded to keep the surgeon and animal caretakers blind to the experimental group.

### The rat full thickness MCL tear model

**Figure 1:**
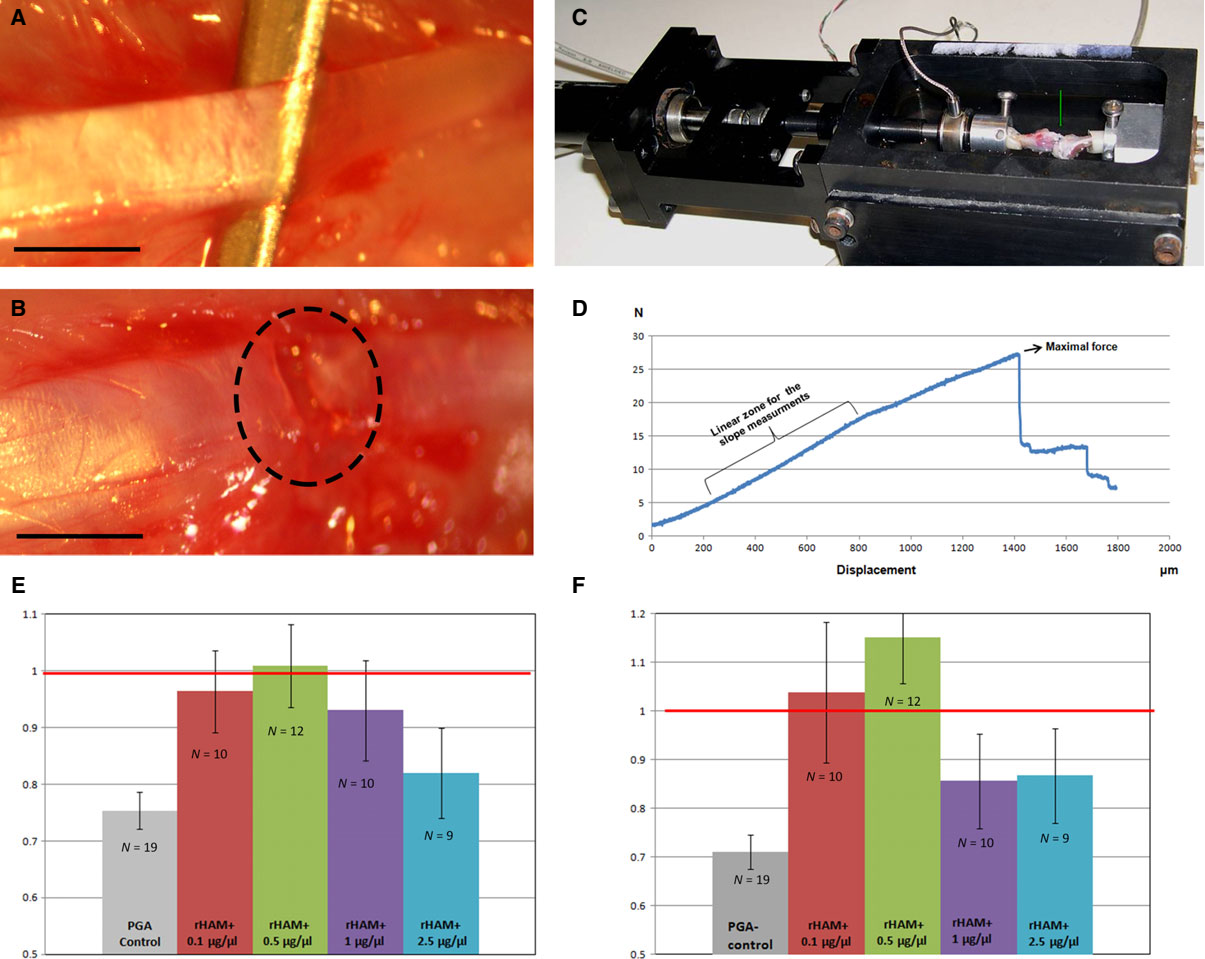
Mechanical properties of healing MCL. (**A**) Intact MCL was elevated on forceps. (**B**) Transected MCL. The site of transection is marked by dotted circle, bar = 1 mm. (**C**) Bone‐ligament‐bone unit fastened in the clamping device which is placed within the materials testing machine. The arrow points to the MCL. (**D**) Force/displacement chart of normal MCL (un‐transected). Maximal force is the force at which the ligament was torn during stretching. Stiffness was calculated from the linear part of the graph. Higher slope represents stiffer ligament. (**E**) Maximal force at failure and (**F**) stiffness were compared between the transected MCL and the un‐transected MCL from the contra‐lateral leg of each rat, after application of 0.1, 0.5, 1, 2.5 μg/μl rHAM ^+^ dissolved in 2.25% PGA carrier (experimental), or 2.25% PGA carrier alone (control). N denotes the number of rats in each experimental/control group.

All rat experiments were approved by Hadassah Medical School Animal Care Ethical Committee, Hadassah ‐ Hebrew University, Jerusalem. 70 adult female Sabra rats weighing about 300 g were used. The surgical procedure was performed under anaesthesia, keeping sterile conditions. Rats were anesthetized with intraperitoneal injection of ketamine hydrochloride (60 mg/kg; Vetoquinol, Lure, France) and xylazine hydrochloride (10 mg/kg; Eurovet, Heusden‐Zolder, Belgium). Subcutaneous tramadol (Grunenthal, Bedminster, NJ, USA) was injected for pain relief. The right knee was approached through a medial skin incision, under dissecting microscope (Zeiss, Göttingen, Germany) the MCL was isolated, raised on scissors and cut transversely (Fig. 1A and B). The wound was rinsed with normal saline. On each day of operation several experimental groups and a control group of animals were operated. In the experimental group, 7 μl of rHAM^+^ dissolved in 2.25% PGA solution was applied over the transection zone, covering the edges of the MCL stumps. In the control group 7 μl of PGA solution alone was applied. The surgeon was blind to the type (and concentration) of the applied solution at the time of surgery and later on. The skin was sutured with a non‐degradable 5‐0 nylon suture (Ethicon, Somerville, NJ, USA). After the operation all animals received tramadol twice, for pain relief during the first day. All animals were monitored for signs of pain and infection. No cast or dressing was applied and the animals were allowed unrestricted cage movement. To follow the course of regeneration rats were killed with an overdose of penthal (CTS, Kyriat Malachi, Israel), 4 days and 12 weeks after operation.

### Mechanical testing

In each rat the right leg (treated) and the left leg (un‐treated) femur‐MCL‐tibia unit were gently isolated under dissecting microscope (Zeiss), such that the MCL remained the only connection between the femur and tibia. The specimen was wrapped in cotton gauze soaked in normal saline solution and stored at −20°C up to testing. On the day of testing, the specimens were thawed in normal saline. The proximal end of the femur and the distal end of the tibia were placed in a cylindrical container, which was filled with poly‐methyl‐methacrylate (PMMA; GC America Inc., Alsip, IL, USA). The potted PMMA was than fastened to a custom‐designed clamping device (Fig. 1C), that was fastened within a custom‐built micro‐mechanical testing device such that one PMMA cylinder was attached to the unmovable wall of the device, while the other PMMA cylinder was attached at no angle to an anvil which was attached in series to a load cell (model 31; Honeywell, Sensotec, Columbus, OH, USA) attached in turn to a high‐precision linear motor (Physik Instrumente GmbH, Karlsruhe, Germany). The experiments were conducted while the sample was immersed in saline, at room temperature. The ligaments were distracted along a linear path at a constant rate of 16.6 μm/sec. under displacement control for a total distance of 3 mm or until failure, while continuously recording the force and displacement, until complete failure (tear) of the ligament (noted as a sudden decrease in force by at least 50%). Force displacement curves were plotted for each ligament. We chose to focus on the maximal force at failure, which represents the strength of the ligament, and on the slope of the graph at its linear part, which represents the stiffness of the ligament (Fig. 1D). Higher slope means stiffer ligament, while lower slope represent a ligament which is more compliant. Normal, un‐transected MCLs were assessed first, while calibrating the biomechanical tensioning system. The results of the calibrating (left and right) MCLs were in the same range as the results of the left MCL (un‐operated MCL), which was measured and compared to the transected right MCL in each experimental rat. Because of individual differences in mechanical properties, in each rat the two parameters measured for the right, transected MCL were divided by the same properties of the left, normal, un‐transected MCL. A ratio of 1.0 represents complete return to the original mechanical properties.

### Preparing, sectioning and analyses of the ligaments by histological, scanning electron microscopy and immunohistochemical methodologies

#### Paraffin sections for haematoxylin and eosin staining, picrosirius red staining and indirect immunohistochemistry

Twelve weeks after transection both the right and left MCL were harvested. The MCLs were fixed in 4% para‐formaldehyde for 24 hrs at 4°C, dehydrated in increasing concentrations of ethanol, embedded in paraffin and sectioned (5 μm thick). Six sections from each ligament of two rats from every experimental group, treated with various concentrations of rHAM^+^ dissolved in PGA carrier (0.1, 0.5, 1 μg/μl rHAM^+^) and from the control group treated only with PGA carrier, were stained with haematoxylin and eosin (Bio Optica, Milano, Italy; Surgipath Medical Industries, Richmond, IL, USA) and three slides were stained with picrosirius solution (Sigma‐Aldrich, St. Louis, MO, USA). All slides were examined by Axioskop (Zeiss); haematoxylin and eosin by un‐polarized light and picrosirius red by polarized light. Pictures were taken using ProgRes C10 (Jenoptik, Jena, Germany).

Nine sections from each ligament of the above experimental groups, treated with various concentrations of rHAM^+^ dissolved in PGA carrier, and from the control group treated only with PGA carrier, were analysed for collagen I, collagen III and neurofilament expression using indirect immunohistochemistry (see section 2.5 below).

#### Scanning electron microscopy

Twelve weeks after transection, both the right and left MCL from two rats treated with 0.5 μg/μl rHAM^+^ dissolved in PGA (experimental), and two rats treated only with PGA (control), were harvested. The MCLs were fixed with Karnovsky's fixative (2% PFA, 2.5% Glutaraldehyde in 0.1 M Cacodylate buffer, pH = 7.4) for 4 hrs at room temperature, followed by 1/2 diluted Karnovsky's fixative overnight at 4°C. Samples were post‐fixed in 2% OsO_4_ in 0.1 M cacodylate buffer for 1 hr, and dehydrated in a graded series of alcohols (from 30% to 100%). Following dehydration by alcohols samples were dried in CPD (K850; Quorum Technology, Laughton, UK), sputtered by Gold, and examined with FEI Quanta 200 scanning electron microscopy (FEI, Hillsboro, OR, USA).

#### Frozen sections for indirect immunohistochemistry and immuno‐fluorescence

Four days after transection, both the right and left MCL of 4 rats treated with 0.5 μg/μl rHAM^+^ dissolved in PGA carrier, and 5 rats treated only with PGA carrier, were harvested. The MCLs were fixed in 4% para‐formaldehyde for 24 hrs at 4°C, and incubated in 30% sucrose solution at 4°C until the tissue soaked in the tube. The tissues were then embedded in O.C.T compound (Sakura Fintek Inc, Torrance, CA, USA), frizzed to −80°C and cryo‐sectioned (Leica Biosystems, Wetzlar, Germany; 8 μm thick). Indirect immunohistochemistry was performed using anti CD105 antibody (Abcam plc, Cambridge, UK), and immuno‐fluorescence using anti STRO‐1 (R&D Systems Inc., Minneapolis, MI, USA) and anti Ki67 antibody (Bioscience Inc., San Diego, CA, USA).

### Indirect immunohistochemistry

Indirect immunohistochemical analyses for detection of collagen I and collagen III expression was performed on 9 paraffin sections from each ligament. Two MCLs were analysed from each experimental group, 12 weeks after treatment with; 0.1, 0.5, 1 μg/μl rHAM^+^ dissolved in PGA carrier, only PGA carrier and untreated normal ligament. Detection of free nerve endings was performed on three MCLs from each group: experimental‐ treated with 0.5 μg/μl rHAM^+^ dissolved in PGA carrier, control‐ only PGA carrier, and untreated normal ligament.

Paraffin slides were deparaffinized, hydrated, rinsed in PBS and endogenous peroxidase activity was blocked by 3% H_2_O_2_ (diluted in methanol) for 10 min. Proteinase K antigen retrieval (BioGenex, San Ramon, CA, USA) was performed for detection of collagen I, and Uni‐trieve (INNOVEX Biosciences Inc., Richmond, CA, USA) antigen retrieval – for collagen III, both for 30 min. at 60°C. No antigen retrieval was used for detection of the free nerve endings.

CD105 expression was evaluated on cryo‐sections prepared from ligaments 4 days after treatment with 0.5 μg/μl rHAM^+^ dissolved in PGA carrier (4 rats) or PGA carrier alone (5 rats). The O.C.T. compound from cryo‐sections was rinsed with PBS solution and incubated for two hours in 75 mM ammonium chloride to reduce the tissues auto‐fluorescence. Slides were rinsed in PBS and endogenous peroxidase activity was blocked by 3% H_2_O_2_, diluted in methanol, for 10 min. No antigen retrieval was used for detection of CD105.

Slides were blocked using background buster (INNOVEX Biosciences Inc.), peptide blocking agent for background removal, for 30 min., followed by over‐night incubation in the first antibody (diluted in PBS) at 4°C in a humidified chamber. The primary antibodies used were: (*i*) a mouse monoclonal antibody against the full length collagen I, purified from cow, cross‐reacting with rat and other species (ab90395; Abcam), diluted to 1/200; (*ii*) a rabbit polyclonal antibody against the full length native collagen III (purified) corresponding to amino acids 1‐1466, cross‐reactivity with rat and other species (ab7778; Abcam), diluted to 1/100; (*iii*) a mouse monoclonal antibody against rat neurofilaments, cross‐reactivity with mouse, chicken and human (ab24574; Abcam), diluted to 1/500; and (*iv*) a rabbit polyclonal antibody against a synthetic peptide corresponding to the C‐terminal region of human CD105, cross‐reactivity with rat and other species (ab107595; Abcam), diluted to 1/100. After rinsing, collagen I and anti‐neurofilaments slides were treated with HRP‐polymer antimouse (ZUC050‐006; Zytomed Systems, Berlin, Germany) for 30 min. Collagen III and CD105 slides were treated for 30 min. with HRP‐polymer anti‐rabbit (ZUC032‐006; Zytomed Systems). All slides were counter stained with haematoxylin and examined by Axioskop (Zeiss). Pictures were taken using ProgRes C10 (Jenoptik).

### Immuno‐fluorescence

STRO‐1 and Ki67 expression were evaluated on cryo‐sections prepared from MCLs 4 days after treatment with 0.5 μg/μl rHAM^+^ dissolved in PGA carrier (4 rats), or PGA carrier alone (5 rats).

The O.C.T. compound was rinsed with PBS solution and incubated for two hours in 75 mM ammonium chloride to reduce the tissues auto‐fluorescence. For detection of Ki67 expression membrane permeabilization was performed by incubation in 0.25% Triton X100 (JT Bakerinc, Phillipsburg, NJ, USA) for 30 min. All slides were blocked using background buster (INNOVEX Biosciences Inc.) for 30 min., followed by over‐night incubation in the first antibody (diluted in PBS) at 4°C in a humidified chamber. The primary antibodies used were: (*i*) a mouse monoclonal antibody against human STRO‐1, diluted to 50 μg/μl (MAB1038; R&D Systems, Inc.), (*ii*) a rat monoclonal antibody against human Ki67, conjugated to FITC, cross‐reactivity with rat and other species. Diluted to 1/50 (11‐5698e; Bioscience Inc.). After rinsing, the STRO‐1 sections were incubated for two hours with the secondary antibody Alexa fluor 488 goat antimouse IgG, diluted in PBS to 1/200 (A11001; Invitrogen, Calsbad, CA, USA). Cell nuclei were counter stained with 4′,6‐diamidino‐2‐phenylindole, dihydrochloride (DAPI; KPL, Gaithersburg, MD, USA). Slides were examined by a confocal laser microscope LSM710 (Zeiss).

### Quantification of CD105, STRO‐1 and Ki67 positive cells for statistical analyses

Four rats were treated with 0.5 μg/μl rHAM^+^ dissolved in PGA, and five were treated with PGA carrier alone.

For quantification of CD105 expression 6 slides were stained from each ligament, and 5 photographs were taken from each slide. All positive cells (brown staining) were counted in each photograph. The researcher was blind for the treatment the rat received. The mean value of positive cells for each ligament was calculated.

For quantification of STRO‐1 and Ki67 expression, 3 slides were stained from each ligament, and 7 photographs were taken from each slide. In each photograph, the area stained by the antibody was divided by the number of stained nuclei. The fluorescent signals were measured using Image Pro Analyzer 7.0 software (Media Cybernetics Inc., Rockville, MD, USA). The researcher was blind for the treatment the rat received. The mean value of positive staining per number of nuclei was calculated for each ligament.

### Statistical analyses

Statistical analysis of mechanical measurements was performed by comparing the different experimental groups (five concentrations of rHAM^+^ dissolved in PGA carrier) to the control (PGA carrier alone), using the anova test, with *post hoc* tests and the Dunnett's correction of the significance level for multiple pairwise comparisons.

Statistical analysis of CD105, STRO‐I and Ki67 expression was performed by comparing the mean values of the experimental and control groups (pairwise comparison) using the a‐parametric Mann–Whitney test. *P*‐values <0.05 were considered as statistically significant.

## Results

### Mechanical measurements

Full thickness MCL tear model (Fig. 1A and B) was performed in the right knee of rats. In the experimental groups 0.1, 0.5, 1 or 2.5 μg/μl rHAM^+^ dissolved in PGA carrier was applied, while in the control group PGA carrier alone was applied. 12 weeks after transection, isolated MCL of both knees, attached to the femur and tibia, was connected to a custom‐built micromechanical materials testing machine (Fig. 1C), which allows continuous recording of force and displacement until failure (tear) of the ligament (Fig. 1D). In each rat the ultimate force and ligament stiffness of the right, transected MCL were compared to the left, un‐transected MCL, such that a value of 1.0 represents complete return to original strength and stiffness. Histograms of mechanical properties (Fig. 1E and F) show that a dose dependent significant healing process was induced by rHAM^+^. Treatment with PGA carrier alone (control) yielded on average 25% weaker and 30% more lax ligaments as compared to untreated ligaments. At concentrations of 0.1–0.5 μg/μl rHAM^+^ there was an increase in the mechanical properties of the ligaments. Treatment with 0.1 μg/μl rHAM^+^ yielded stronger, and significantly stiffer ligaments (*P* = 0.029) than treatment with PGA alone, while application of 0.5 μg/μl rHAM^+^ yielded the best results; transected ligaments were on average as strong as the normal un‐transected ligaments (*P* = 0.014) and even stiffer (*P* < 0.01) than un‐transected MCL. Treatment with 1 and 2.5 μg/μl rHAM^+^ yielded almost no effect on the mechanical properties as compared to PGA‐treated ligaments.

### Ligament structure and composition

**Figure 2:**
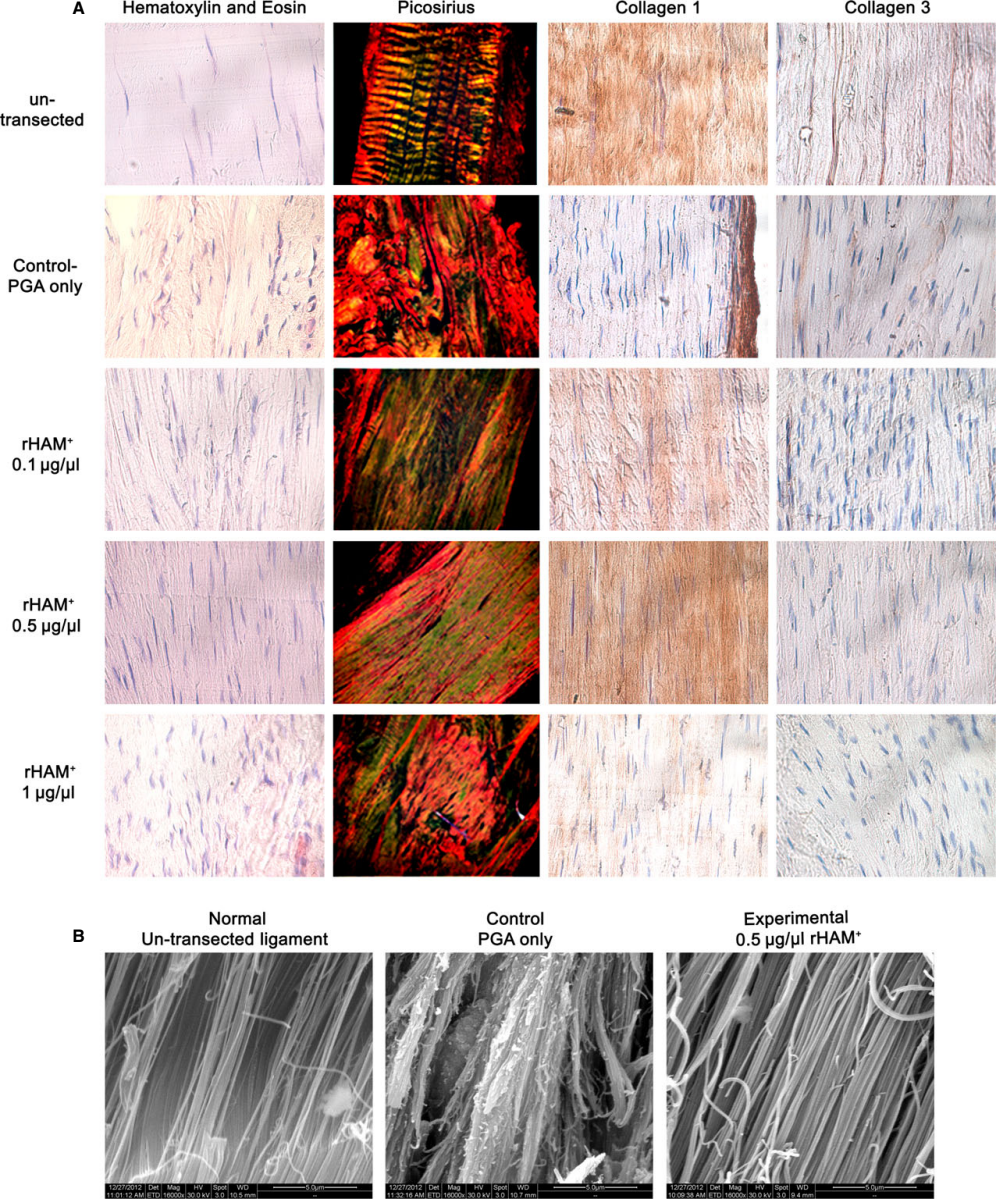
Morphometric and composition analyses of the transected area 12 weeks after operation. (**A**) Haematoxylin and eosin staining, picrosirius red staining and immunohistochemistry using collagen I and III antibodies (brown staining) of un‐transected ligaments, transected area of experimental ligaments treated with 0.1, 0.5, 1 μg/μl rHAM ^+^ dissolved in PGA, and transected area of control ligaments treated with PGA carrier alone. (**B**) Scanning electron microscope pictures of ligaments 12 weeks after operation: left ‐ un‐transected ligament, middle – transection zone of a ligament treated with PGA carrier (control), right – transection zone of a ligament treated with 0.5 μg/μl rHAM ^+^ dissolved in PGA (experimental), magnification ×16,000.

Structure and composition of ligaments was studied using histology, electron microscopy and immunohistochemical reactions twelve weeks after transection and application of various concentrations of rHAM^+^ dissolved in PGA (experimental), or PGA carrier alone (control) (Fig. 2). Staining with haematoxylin and eosin and with picrosirius red (specific staining for collagen fibres) showed that after application of 0.5 μg/μl rHAM^+^ the arrangement of fibres along the direction of forces of the MCL was similar to that of normal un‐transected MCL, although the recovered area was more cellular than the normal un‐transected ligament (Fig. 2A). Horizontal striation of un‐transected ligament is most probably because of the preparation. Similar, but less prominent results were obtained using 0.1 μg/μl rHAM^+^, in which the fibres of the healed MCL were also arranged along the direction of forces. After application of 1 μg/μl rHAM^+^, the fibres at the transected area seemed less organized, and resembled control, PGA‐treated ligaments, where the fibres were not arranged and tissue structure resembled scar tissue (Fig. 2A).

Collagen I is the major protein in ligaments which provides the mechanical properties and strength of normal ligaments. Immuno‐histochemical analyses showed that 12 weeks after transection and application of 0.5 μg/μl rHAM^+^, mature collagen I content was similar to un‐transected MCL. At concentration of 0.1 μg/μl, collagen I expression was much higher than in PGA‐treated MCL, while at 1 μg/μl rHAM^+^, collagen I expression resembled treatment with PGA (Fig. 2A). These results were in‐line with the mechanical force measurements that demonstrated a bell shaped behaviour at different rHAM^+^ concentrations.

Twelve weeks after transection, no significant change in expression levels of collagen III between un‐transected ligament, PGA control and different experimental groups was detected (Fig. 2A), indicating the healing process reached the maturation stage.

Pictures of the transection zone, obtained by scanning electron microscope 12 weeks after operation (Fig. 2B), showed that after treatment with 0.5 μg/μl rHAM^+^, ligament fibres were arranged in an elongated orientation, similar to un‐transected ligament. In the control, treated with PGA carrier, there was no spatial arrangement of fibres, similar to scar tissue.

### Proprioceptive innervation of the ligaments

**Figure 3:**
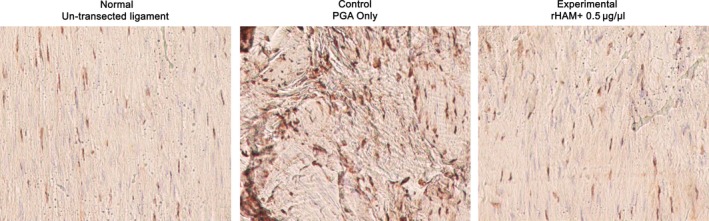
Immunohistochemistry using a mouse monoclonal antibody against rat neurofilaments (brown staining) of un‐transected ligaments (left), transected area 12 weeks after the operation of experimental ligament treated with 0.5 μg/μl rHAM ^+^ dissolved in PGA (right) and of control ligament treated with PGA carrier alone (middle).

The arrangement of neurofilaments was studied using immunohistochemistry (Fig. 3). In the normal un‐transected ligaments the neurofilaments were arranged along the collagen I fibres, parallel to the direction of force. Similar arrangement was seen in the experimental ligaments treated with 0.5 μg/μl rHAM^+^. In the control MCL, treated only with PGA carrier, there was no spatial arrangement of collagen fibres and of the neurofilaments.

### Cellular mechanisms associated with the healing process of the MCL

**Figure 4:**
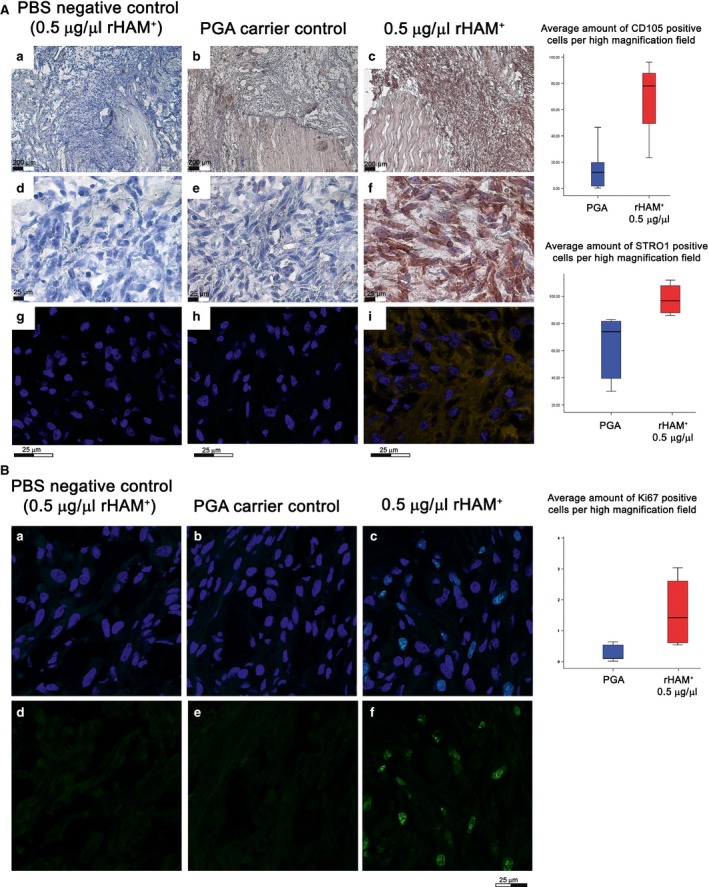
Expression of MSC markers CD105 (**a**–**f**) and STRO‐1 (**g**–**i**) in the arranging haematoma 4 days after transection: **a**–**f**) Immunohistochemistry showing the expression of CD015 (brown staining); **a**–**c** dotted yellow line marks the edges of the stumps of transected ligaments. The tissue filling the gap between ligament stumps was inflammatory tissue. **d**–**f** magnifications of the inflammatory tissues, marked by black squares, in **a**–**c**. (**g**–**i**) Immuo‐fluorescence analysis showing the expression of STRO‐1 (yellow). Cell nuclei were stained with DAPI (blue). (**a**,** d** and **g**) Negative control for the immunological reaction; ligaments treated with 0.5 μg/μl rHAM ^+^, incubated with PBS instead of antibody against CD105 (**a** and **d**) or STRO‐1 (**g**). (**b**,** e** and **h**) Control – application of PGA carrier alone. (**c**,** f** and **i**) Experimental – application of 0.5 μg/μl rHAM ^+^. (**b**) Immuo‐fluorescent evaluation of cell proliferation using Ki67 (green) in the arranging haematoma 4 days after transection. Cell nuclei were stained with DAPI (blue). (**a** and **d**) Negative control for the immuo‐fluorescence reaction; incubation with PBS instead of the Ki67 antibody. (**b** and **e**) Control ‐application of PGA carrier alone. (**c** and **f**) Experimental – application of 0.5 μg/μl rHAM ^+^.

Formation, remodelling and regeneration of tissues generally require recruitment of multipotent cells. We studied the cell population at the arranging haematoma between the transected stumps. Four days after transection, significantly more cells expressing the MSC markers CD105 (*P* < 0.01) and STRO‐1 (*P* < 0.032) 9, 10 were detected in ligaments treated with 0.5 μg/μl rHAM^+^, as compared to treatment with PGA carrier alone (Fig. 4A), while after application of 1 μg/μl rHAM^+^, no difference in the amount of MSC was detected as compared to PGA (data not shown).

The increase in number of cells expressing MSC markers in the experimental ligament could be because of cell recruitment, cell proliferation, or both. To test whether the enrichment of MSC is caused by cell proliferation, the expression of Ki67, a proliferation marker, was studied. Treatment with 0.5 μg/μl rHAM^+^ caused significant cell proliferation at the region between the transected stumps 4 days after transection, as compared to treatment with PGA carrier (*P* < 0.032) (Fig. 4B).

## Discussion

Ligaments do not possess native regeneration capabilities. In response to tear, ligaments produce scar tissues which are significantly inferior in their mechanical properties, lack correct proprioceptive innervation, and usually do not clinically suffice.

We have previously shown that application of the recombinant human amelogenin (rHAM^+^) alone, produced in our laboratory 7, brought about regeneration of the tooth‐supporting tissues; alveolar bone, PDL and cementum, after induction of chronic experimental periodontitis in a dog model 8. The results presented here show that a single application of rHAM^+^ caused significant functional and structural healing of skeletal ligaments, instead of formation of unorganized, less functional, fibrous tissue which characterize the normal healing process of ligaments. Treatment with PGA carrier alone (control) yielded on average 25% weaker and 30% more lax ligaments as compared to untreated ligaments 3 months after operation. However, treatment with 0.1–0.5 μg/μl rHAM^+^ induced significant healing of torn MCL, since the mechanical properties improved as compared to control ligaments. After treatment with 0.5 μg/μl rHAM^+^, the healed ligaments were on average as strong as the normal un‐transected ligaments and even stiffer, suggesting regeneration has occurred. In clinical practice, mild stiffness of ligaments can be treated with physiotherapy, while there is no satisfactory solution for lax ligaments beside surgical replacement. Furthermore, the arrangement of the neurofilaments along the collagen fibres and parallel to the direction of the force, suggests that the proprioceptive sensation was also recovered. Treatment with 1 μg/μl rHAM^+^ and 2.5 μg/μl rHAM^+^ yielded almost no effect on the mechanical properties as compared to the control group treated only with PGA. Although in human torn MCL usually does not require any surgical intervention, for having satisfying clinical results because of the effect of other knee stabilizers, this model serves as a proof of concept for the regeneration capabilities of rHAM^+^, which could be utilized for regeneration of other skeletal ligaments.

The spatial arrangement of collagen fibres and the quantity of collagen I, which gives the ligament its strength, were also similar when comparing transected MCL treated with 0.5 μg/μl rHAM^+^ to the un‐transected ligaments, while no such arrangement was observed in the control (treated with PGA carrier) ligaments, in which scar tissue was produced. Similar, but less prominent results were obtained using 0.1 μg/μl rHAM^+^, in which the fibres of the regenerated MCL were also arranged along the direction of forces. Interestingly, at a concentration of 1 μg/μl rHAM^+^, just twice the concentration that yielded the best regenerative effect, it seems that the beneficial effect of rHAM^+^ was inhibited. Previous cell culture studies also described a concentration dependent effect of amelogenin. Huang *et al*. 11, tested the effect of adding 1–1000 ng/ml rh174, another recombinant human amelogenin protein, on the proliferation of MSC culture. They found an increase in the effect of rh174 up to a concentration of 100 ng/ml, with a decrease in activity at 1000 ng/ml.

Formation, remodelling and regeneration of tissues generally require recruitment of multipotent cells. Mesenchymal stem cells (MSCs) are able to differentiate into ligament, tendon, bone, cartilage, muscle, fat and other connective tissues. Not only do MSCs have the capacity to differentiate into various types of cells, depending on the tissue matrix, they also actively contribute to their milieu by secreting soluble products that participate in MSC and surrounding cell phenotype 12. At sites of injury these cells are released, where they secrete large quantities of bioactive factors that are both immuno‐modulatory and trophic. The trophic activity inhibits ischaemia‐caused apoptosis and scarring while stimulating angiogenesis and the mitosis of tissue intrinsic progenitor cells 13. Our results show that 4 days after injury and application of 0.5 μg/μl rHAM^+^, there was a significant increase in the amount of cells expressing the MSC markers CD105 and STRO‐1 at the injured site surrounding the ligament stumps. On the other hand, after application of 1 μg/μl rHAM^+^, there was no difference in the amount of MSC at the regenerating area as compared to treatment with the control – PGA carrier alone (data not shown). The ability/inability to increase the amount of MSC 4 days after transection, correlated with the mechanical and structural properties 3 months after transection. Hence, it seems reasonable to conclude that the rich MSC population in the region of the arranging haematoma between the stumps of the transected ligament enabled the oriented structural and mechanical repair of the MCL, rather than the normal healing process producing scar tissue.

The increase in number of cells expressing MSC markers in the experimental ligament could be because of cell recruitment, cell proliferation or both. The increased cell proliferation at the injured site 4 days after transection and treatment with 0.5 μg/μl rHAM^+^ may partially explain the increase in MSC content. Li *et al*. 14, described significant enhancement of proliferation of PDL fibroblasts *in vitro*, after application of 10 μg/ml of recombinant porcine amelogenin to the culture. On the other hand, our previous organ culture experiments showed that agarose beads soaked with rHAM^+^ induced cell recruitment towards the beads, while control beads caused no cell recruitment 15. Hence, the increase in amount of MSC induced by rHAM^+^ might involve recruitment of cells, followed by proliferation.

The limitations of this study are: (*i*) it is mainly a proof of the concept study that aims to explore the ability of rHAM^+^ to cause significant healing of injured skeletal ligaments. Hence, we chose the well studied and accepted MCL tear model, although in most cases the natural scarring of torn MCL is sufficient clinically, and do not need surgical intervention. (*ii*) Furthermore, the cellular and molecular pathways by which application of amelogenin induces the significant healing of transected MCL should be further studied in depth. In spite of these limitations it seems that the accumulating results can be used as a platform for skeletal ligament regeneration. We suggest that application of amelogenin can serve as the biological solution for the most common ligamentous injuries.

## Author contribution

S. Hanhan – Preparation of specimens for the mechanical force analyses, proprioception analyses. A. Ejzenberg – CD105, STRO1 and Ki67 expression analyses. K. Goren‐ Preparation of specimens for the mechanical force analyses. F. Saba‐ Tissue preparation and assistance in the histological analyses. Y. Suki‐ Tissue preparation and assistance in the histological analyses. S. Sharon‐ Assistance in planning the research. D. Shilo‐ Assistance in tissues preparation. J. Waxman‐ Immunohistochemical analyses of collagen expression. E. Spitzer‐ Assistance in surgical procedures. R. Shahar‐ The designer of the mechanical force equipment in charge of designing and adjustment of measurement method. A. Atkins‐ Assistance in the mechanical measurements. M. Liebergall‐ Head of the orthopaedic complex at Hadassah Medical Centre, assisted in the study design. A. Blumenfeld‐ Part of the team that designed and planed the research helped guiding the students during the work and writing this manuscript. D. Deutsch‐ Head of the dental research laboratory where this study was conducted. A. Haze – Primary investigator, study designer and the main surgeon.
